# The Bristol Hip View: Its Role in the Diagnosis and Surgical Planning and Occult Fracture Diagnosis for Proximal Femoral Fractures

**DOI:** 10.1155/2013/703783

**Published:** 2013-01-08

**Authors:** J. Harding, T. J. S. Chesser, M. Bradley

**Affiliations:** ^1^Departments of Radiology, Frenchay Hospital North Bristol NHS Trust, Bristol BS16 1LE, UK; ^2^Departments of Trauma and Orthopaedic Surgery, Frenchay Hospital North Bristol NHS Trust, Bristol BS16 1LE, UK; ^3^Departments of Orthopaedics, Frenchay Hospital North Bristol NHS Trust, Bristol BS16 1LE, UK

## Abstract

*Aim*. To evaluate whether a modified radiographic view of the femoral neck improves the diagnosis of occult proximal femoral. *Materials and Methods*. Prospective study of patients presenting with clinically suspected proximal femoral fractures or who underwent traditional plain radiographic views and the Bristol hip view (a 30-degree angled projection). Six blinded independent observers assessed the images for presence of a fracture, anatomical level, and displacement. *Results*. 166 consecutive patients presenting with the clinical diagnosis of a proximal femoral fracture, of which 61 sustained a fracture. Six of these were deemed occult due to negative plain and had proven fractures on subsequent cross-sectional imaging. The Bristol hip view demonstrated five of these six fractures. It performed better than the traditional lateral hip view to identify the injury. The Bristol hip view predicted correctly the fracture type and displacement in all cases and missed only one of the occult fractures. *Conclusion*. The Bristol hip view is more sensitive and clearer than a lateral projection for patients. It adds useful diagnostic information and performs better than the traditional views in occult fractures. Its use may prevent the need for further cross sectional imaging and subsequent surgical delay.

## 1. Introduction

There is a recent impetus in the early diagnosis and treatment of hip fractures [1]. The incidence of occult femoral neck fractures on plain radiographs is reported between 1% and 4% but this figure is higher in selected study groups [2–5]. Little has been published regarding optimising plain radiography to increase sensitivity and decrease the incidence of occult femoral neck fractures. Such an approach would help obviate the need for expensive additional imaging investigations. The standard radiographic views for the patient with a suspected femoral neck fracture have traditionally been an anteroposterior (AP) pelvis with lateral hip view.

**Figure 1:**
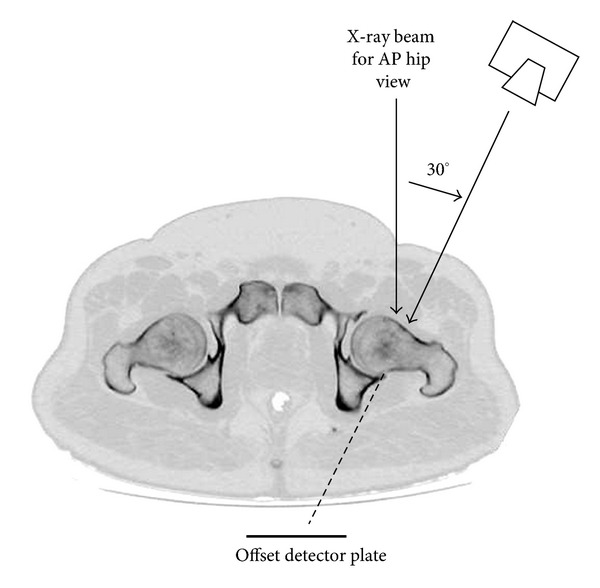
X-ray tube is angled 30° from the vertical, centered on the femoral head with the detector plate offset so that the whole femoral neck and acetabulum are included in the field.

Previously, we have suggested a new radiographic projection (Figure 1) for femoral neck fractures in an experimental study (the Bristol hip view) [6]. The Bristol hip view is obtained with 30° tube angulation, the angle of incidence to the femoral neck being nearer to 90°, and has been shown to demonstrate femoral neck fractures with greater clarity in both the subcapital and mid-cervical regions than on a standard AP view [6].

In this study, the aim was to assess the relative sensitivity and specificity of all the three of these radiographic views and in particular assess whether the Bristol hip view added information leading to the diagnosis of otherwise occult fractures of the neck of femur.

Data was collected surrounding the clinical impact of the Bristol hip view, to establish whether the view allows better anatomical description of any fracture, its displacement, and whether it assists in surgical treatment planning.

## 2. Materials and Methods

**Figure 2:**
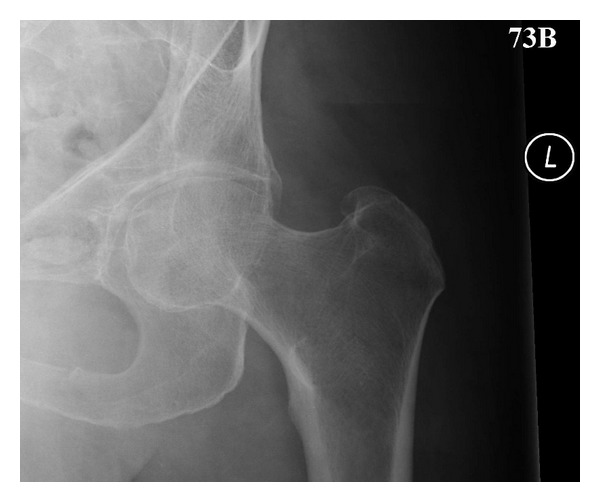
Example of the Bristol hip view—no fracture.

The study was granted approval by the local research ethics committee. The Bristol hip view (Figure 2) was added to the standard views for suspected fractured neck of femur in patients presenting to the emergency department (ED). Patients are imaged on the ED trolley in the same position as the AP view. A standard 30 × 24 cm cassette (landscape) was used, with the beam angled 30° from vertical towards the midline on the symptomatic side, centred on the hip joint, with the beam coned to the size of the cassette and the cassette displaced appropriately by the radiographer in the cassette tray. Exposures are manual and equivalent to the standard AP view (30–40 mAs, 75 kV). Patients are not imaged on the X-ray tables due to their fixed grid running perpendicular to the cassette. This technique does not require movement or rotation of the patient, thus avoiding pain.

Prospectively a series of consecutive patients with clinically suspected fracture of the neck of femur presenting to the ED over a nine-month period were included. Patients were followed up for three months from presentation to allow for any further development of relevant symptoms and ensure accuracy of occult fracture diagnosis.

Patient outcomes were classified as either “fracture” or “no fracture.” The fracture group was determined on the basis of:

- unequivocal plain film evidence at the presenting episode,
- additional imaging (plain radiography, CT, MRI, and isotope bone scan) had been performed and reported as positive for fracture, or
- the patient had definitive surgery for fractured neck of femur during the clinical episode.

Those included under (b) were designated as “occult fractures” for the purposes of the study. The “no fracture” group had no evidence of fracture on initial plain radiography or on any subsequent imaging and were discharged from hospital without surgery (whether from the ED or elsewhere).

166 patients met the inclusion criteria in the study period. Of these, 61 were in the fracture group of which 6 (9.8%) were “occult.” For blind assessment of the films, the 61 patients were balanced for image viewing with 39 randomly selected patients from the “no fracture” group. Accordingly, 300 images were coded with a number and letter (A, B, C-AP, Bristol hip view, and lateral, resp.) and saved from the trust PACS system as standard digital image files using a web PACS application.

Images were labelled with a single experimental code identifier but all other identifying information was removed from images; side markers were permitted but any information added to the original images by radiographers was removed. These images were made available to observers and viewed on standard PC monitors in the normal viewing conditions for clinicians in the ED.

Images were supplied to six assessors, three radiologists with musculoskeletal imaging interest and three experienced Consultant orthopaedic surgeons actively participating in the trauma team. All observers viewed images independently in a randomised order and completed the same response form: each image was viewed in isolation and judged as “fracture” or “no fracture.” When judged a fracture, the observers also recorded the anatomical level (subcapital, basicervical, or pertrochanteric) and whether the fracture was displaced or not. These are all key features for orthopaedic surgeons in deciding their approach to any surgical management.

## 3. Results

166 consecutive patients demonstrated 61 proximal femoral fractures with 105 not sustaining a fracture; the latter were discharged and did not represent in the 3 month follow up period. There were 21 pertrochanteric fractures and 40 neck of femur fractures (Table 1).

For the six fractures designated “occult,” the standard AP and lateral views were either negative for fracture or suspicious and they all underwent collaborative imaging (3 MRIs, 2 CTs, and 1 isotope bone scan). All the observers confirmed fractures on the Bristol hip view in four of the six occult fractures, four of the six observers in one case, and two of the six in one case.

The lateral view is traditionally the most technically challenging view to obtain and this was highlighted in this study; incomplete fracture data was obtained in 20 of the 40 fracture neck of femur patients; this was due to radiographic positioning in frail/sick patients, exposure, and limitation of utility of the digital radiography system. Thus from the lateral view, only 21/40 showed the fracture and 12/40 confirmed the displacement. The Bristol hip view demonstrated the fractures in all cases. It was felt that this view accurately predicted displacement in comparison to the AP view and performed better than the lateral, mainly due to the failure rate of the lateral view. Only in one patient was it felt the AP showed the displacement more accurately.

Treatment involved either fixation with cannulated screws or sliding hip screws. Displaced intracapsular fractures were treated with replacement arthroplasty. One patient died before surgery could be undertaken.

## 4. Discussion

**Figure 3:**
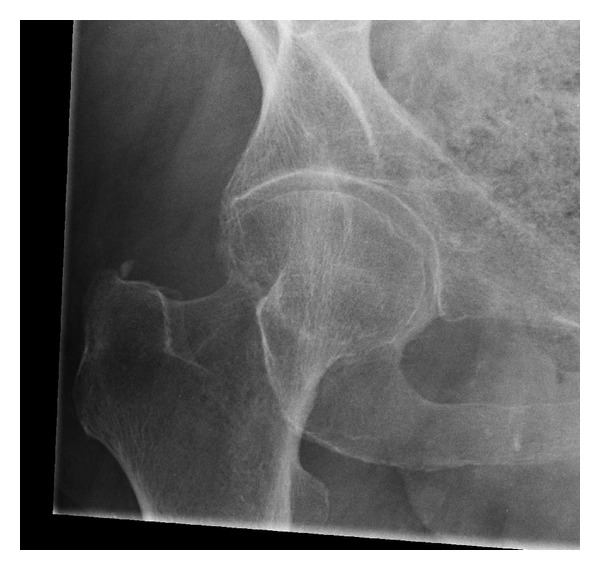
Example of a femoral neck fracture shown on the Bristol hip view.

The Bristol Hip view is modified from the angled view already performed routinely for surgical planning in the assessment of acetabular fractures (the Judet obturator oblique view). The concept is that this angled view should reflect a truer and more reproducible right angle of X-ray beam incidence to the femoral neck and thus demonstrate the anatomy and fractures more clearly (Figure 3). This study highlights how the lateral may often fail to be optimal and thus often noncontributory to the diagnosis and planning. It was shown that half of the laterals in the fracture neck of femur group gave incomplete information. This was due partly to the exposure factors which are dictated by the digital radiography system but also radiographic positioning difficulties in this elderly sick population who are immobile with a leg deformity and often obese. There has been an article suggesting the lateral view is of little use in this population with its only use in detecting displacement of a subcapital fracture which appears displaced on the AP view [7]. It is now accepted that if the fracture is displaced on either view then replacement arthroplasty is recommended rather than internal fixation, so this displacement is important to diagnose as it dictates alternative treatment [1].

**Figure 4:**
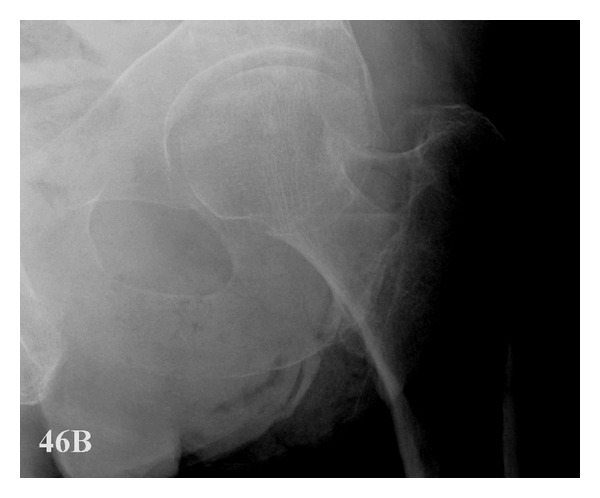
Bristol hip view demonstrating the importance of correct tube angulation. Over angulation of the beam means the fracture could be confused with the posterior border of the acetabulum.

The Bristol hip view also needs care to reproduce accurately the correct positioning; but overall it appears more reproducible than the lateral. Too much angulation may lead to the neck not appearing sufficiently elongated and thus the posterior rim of the acetabulum may mask the fracture (Figure 4). The Bristol hip view was originally described by Bradley et al. using an average femoral neck anteversion angle derived from a combination of CT and historical data, so errors in angulation may also occur due to differences in the anteversion angle as well as the positioning of the leg due to the fracture. In the authors' opinion the success of the Bristol hip view lies with the way it elongates the anatomy of the femoral neck, similarly achieved when investigating the scaphoid fracture with the utilisation of the elongated view along the long axis of the bone.

Regarding the “occult” fractures, the lateral view did not identify any of fractures, whereas the AP was suspicious in two of the six cases. The Bristol hip view however confidently diagnosed four, reported by all the six observers. One fracture was reported by four observers and one by only two. This was due to overangulation of the X-ray tube rendering the posterior wall of the acetabulum over the fracture. They were all correctly reported on subsequent imaging but the Bristol hip view appears to have good potential for occult fracture diagnosis and may therefore obviate the need for further expensive imaging and avoid delays in surgical management whilst waiting for these investigations. Current recommendations are for patients to undergo magnetic resonance scanning for the diagnosis of occult hip fractures; however this is not routinely accessible out of hours, carries additional cost, and can be difficult in those with cognitive impairment [1].

Limitations of the study include the low numbers of occult fractures which precludes calculation of statistical significance. To achieve significance a sample size of over 3000 patients would be required. For displacement, although the study did not have a gold standard against which the criterion was measured, the AP and Bristol Hip view were both comparable, with the lateral view preforming poorly. This is increasingly important due to a change of surgical practice, where displaced fractures are now undergo replacement arthroplasty rather that fixation.

## 5. Conclusion

In the acute setting, for relatively little additional financial cost, time, and radiographic resource, the Bristol hip view adds useful diagnostic information with significantly greater clarity than the traditional lateral view. The Bristol hip view may be of added clinical utility as an additional film, particularly when the optimal lateral view is not obtainable.

The Bristol hip view may perform better than the traditional two views in the setting of occult fractures; however, any statistical significance of this effect in this study is hampered by low numbers.

## Figures and Tables

**Table 1:** Shows the distribution of femoral neck fractures with their displacement.

	Neck of femur fracture	
	Subcapital	Basicervical
Total	16	24
Displaced	13	19
Nondisplaced	3*	5^*∧*^

*3 occult fractures in this group.

^∧^3 occult fractures in this group.
